# Unilateral Triple Canal Repositioning Maneuver: Principles and Design

**DOI:** 10.3390/audiolres15030055

**Published:** 2025-05-08

**Authors:** Marcello Cherchi

**Affiliations:** Department of Neurology, University of Chicago Medicine, 5841 South Maryland Avenue, Chicago, IL 60637, USA; marcello.cherchi@bsd.uchicago.edu; Tel.: +1-‭(773)-834-0323

**Keywords:** vestibular, ocular motor, benign paroxysmal positional vertigo, therapy, treatment maneuver, simulation

## Abstract

**Background**: Benign paroxysmal positional vertigo is the most common cause of dizziness over the lifespan. Management is complicated by both a diagnostic burden (correctly interpreting specific ocular motor findings) and a therapeutic burden (selecting appropriately targeted treatment maneuvers). **Purpose**: Devise a canalith repositioning maneuver to treat simultaneously benign paroxysmal positional vertigo affecting any combination of semicircular canals on one side. If clinically verified, then this will simplify management. **Research design**: After analyzing the results of a computer simulator applied to several published maneuvers for treating benign paroxysmal positional vertigo, we used basic principles to design a maneuver that simultaneously, for all three semicircular canals on one side, advances otoliths toward the crus of each canal and prevents retreat toward the ampule of each canal and then tested the maneuver in computer simulation. **Study sample**: Not applicable. Intervention: Not applicable. Data collection and analysis: Not applicable. **Results**: We developed a maneuver that computer simulation predicts will successfully treat simultaneously benign paroxysmal positional vertigo affecting any combination of semicircular canals on one side. **Conclusions**: This maneuver should be tested empirically as a standalone maneuver and compared to other maneuvers. Clinical relevance statement: If the efficacy of this maneuver is clinically verified, then it will simplify management by diminishing the diagnostic burden (of determining affected canals) and treatment burden (of selecting the appropriately targeted maneuvers).

## 1. Introduction

Benign paroxysmal positional vertigo (BPPV) is the most common cause of dizziness over the lifespan [1]. Fortunately, it is also one of the most treatable otologic disorders, and the treatment involves a series of canalith repositioning maneuvers rather than medication or surgery. Unfortunately, treatment of BPPV is offered far less than would be appropriate [2] in some cases because the diagnosis is not sought, or when identified, it is not treated [3].

Neuhauser [4] estimated the 1-year prevalence of BPPV to be 1.6% and the 1-year incidence of BPPV to be 0.6%. As of this writing, the US population is about 341 million (https://www.census.gov/popclock/, accessed on 30 April 2025) [5], so currently, about 5.4 million people have BPPV, and in the next 12 months, about 2 million people will develop BPPV.

This population of patients is too large to be seen by the relatively few specialists (audiologists, vestibular physical therapists, neurotologists, otoneurologists) trained to localize (i.e., identify the affected canal or canals) and treat (i.e., select and apply the appropriately targeted therapeutic maneuvers) BPPV. Consequently, these patients’ initial (and sometimes only) medical encounters are usually with front-line providers (primary care physician, emergency room, general physical therapist).

Despite evidence that BPPV can be successfully managed by front-line providers, including primary care physicians [6,7] and emergency physicians [8], there are several barriers to the identification and treatment of BPPV in such settings [9,10]. These barriers include practitioners’ uncertainty about how to test for BPPV, uncertainty in how to interpret any nystagmus elicited by diagnostic maneuvers, uncertainty about what therapeutic maneuvers to apply, and lack of confidence in executing such maneuvers. Given that any combination of six semicircular canals can be affected, the selection of an appropriately targeted maneuver may not be straightforward.

If the diagnostic burden (identifying the affected canals) and treatment burden (selecting the correct treatment maneuvers) could be diminished, then this would simplify the task front-line providers face in caring for this immense population of patients.

Most of the published canalith repositioning maneuvers aim at a single canal. There are exceptions, such as the Brandt–Daroff maneuvers [11] to treat bilateral posterior canals, the Yacovino maneuver [12] to treat bilateral anterior canals, and the Kurtzer hybrid maneuver [13] to treat bilateral horizontal canals. There have been fewer attempts at treating multiple canals on the same side [14].

It has been documented that treatment targeting BPPV in a particular canal can sometimes unintentionally yet successfully treat BPPV in another canal [15]. Thus, it should be feasible to leverage whatever features make that possible and devise a maneuver that intentionally treats multiple canals simultaneously.

We, therefore, sought to develop a canalith repositioning maneuver to treat benign paroxysmal positional vertigo involving any combination of semicircular canals on one side. The rationale is that such a maneuver could provide several advantages. First, it would be unnecessary to know which canal or combination of canals is involved. Second, it could treat multiple canals (on the same side) simultaneously. Third, since it could treat all three canals (on the same side) simultaneously, it would lower the risk of triggering a canal conversion [16,17,18,19,20,21,22,23,24]. Fourth, these features would make it easier and more effective for providers to treat BPPV and would make it easier for patients attempting self-treatment.

To put this in perspective, Shim and colleagues [25] studied 1054 patients with BPPV and reported that 1005 (95.4%) had single-canal BPPV. Only 49 (4.6%) patients had multi-canal BPPV. Of all 49 patients with multi-canal BPPV, 39 (79.6%) had multiple unilateral canal involvement, and of those 39 patients, 31 (63%) exhibited a combination of posterior and lateral canals. The occurrence of unilateral BPPV involving all three canals simultaneously is so rare [26] that it would be nearly impossible to conduct even a small “trial” of a maneuver in treating actual patients, so the only realistic way to study this is through computerized simulation. Practically, however, our purpose in designing a maneuver for treating all three canals in one ear is not because we expect to find such cases; rather, it is so that a single maneuver can treat all canals on one side, irrespective of which canal(s) is involved in a particular case.

## 2. Materials and Methods

We began by choosing a BPPV simulator and using it to test several established maneuvers used for treating BPPV.

Mathematical descriptions of what happens in BPPV can be very elaborate, taking into account properties of the canal (canal radius, duct radius, wall deformability), the endolymph (density, viscosity), and otoliths (shape, size, density) [27], as well as a variety of forces (drag, gravitational force, Coriolis force, centrifugal force, Euler force, and vorticeal forces) [28], and the interactions among the canal, endolymph, and otoliths. To make the computational burden more manageable, the literature describing otolith behavior and semicircular canal fluid dynamics often must make a number of simplifying assumptions [27,29,30,31] and use idealized anatomical models (e.g., in which a semicircular canal is a perfect torus). Simulators are usually built on such idealized models [32].

The biological reality does not conform well to these idealizations in many respects, including even basic geometrical features. First, the semicircular canal lumen is not actually a torus, and its cross-sections are not uniform throughout its course [33]. Second, the canal walls are deformable [34]. Third, the semicircular canals are neither truly orthogonal to each other nor truly planar [35]. Fourth, the mid-sagittal plane forms an angle of approximately 41° with the anterior canal and an angle of approximately 56° with the posterior canal, so the pairs of canals in the so-called RALP (right anterior–left posterior) and LARP (left anterior–right posterior) “planes” are not truly co-planar [36,37]. Fifth, there is anatomical variability from person to person [38]. Sixth, the maximal vestibular response vectors may be distinct from the anatomical canal planes [39].

Bearing in mind these limitations, we elected to use the “BPPV Viewer” simulator developed by Teixido and colleagues [40,41], whose semicircular canal reconstructions were based on data drawn from actual temporal bone specimens.

We used the BPPV Viewer to test multiple published maneuvers, as listed in Table 1 below. There are other maneuvers, but we selected these because they are either common or at least have been relatively well-described. For the simulation of each maneuver, we placed otoliths in all three semicircular canals on the right side and conducted 10 test-run simulations. For almost all maneuvers, we set the initial conditions as the “worst-case scenario”, meaning that the otoliths were in the ampulla. The only exception was for geotropic lateral canal BPPV; in these cases, we placed the otoliths near the crural segment of the apex (see below regarding this terminology).

We had two goals in testing these published maneuvers. Our first goal was to assess the degree of success/failure a given maneuver had for the canal(s) it had been designed to target; in treatment failures, we noted the mechanism of failure. Our second goal was to explore whether each maneuver might (unintentionally) treat BPPV in canals other than that for which the maneuver had been developed.

We discuss the results of the simulator for each maneuver.

The Epley maneuver [42] was designed to treat unilateral posterior canal BPPV. The results of the simulation showed, surprisingly, that the Epley maneuver is very effective at treating all three canals on a given side (efficacies of 100% for the posterior canal, 100% for the lateral canal, and 90% for the anterior canal).

The Semont maneuver [43] was designed to treat unilateral posterior canal BPPV. The results of the simulation showed relatively poor efficacy overall (30% for the posterior canal, 10% for the lateral canal, 0% for the anterior canal). The main reason for treatment failure of the intended canal (posterior canal) was that in position 2; otoliths tend to reflux into the anterior canal and then remain there; less commonly, the otoliths approach the posterior canal’s apex but then retreat.

The Semont Plus maneuver [44,45] was designed to treat unilateral posterior canal BPPV. The results of the simulation showed very good efficacy for the intended canal (80% posterior canal), occasional treatment of the anterior canal (30%), and no efficacy for the lateral canal (0%). Treatment failures of the posterior canal occurred when otoliths refluxed into the anterior canal (10%) or when they approached but then retreated from the apex (10%).

The Foster maneuver [46] was designed to treat unilateral posterior canal BPPV. The results of the simulation showed very good efficacy for the intended canal (80% posterior canal). It also had moderate efficacy for the anterior canal (60%); in the cases where the AC was not treated, the otoliths failed to exit the ampulla. The maneuver only treated the lateral canal in 10% of cases.

The Gans maneuver [47] was designed to treat unilateral posterior canal BPPV. The results of the simulation were surprising. The Gans maneuver had poor efficacy for the intended (posterior) canal (20%). In contrast, it had excellent efficacy (100%) for the lateral canal and very good efficacy (80%) for the anterior canal. The lateral canal otoliths sometimes (10%) refluxed into the posterior canal before resolving; another 10% of the time, they remained in the posterior canal (were not successfully treated).

The modified Epley maneuver [48] was designed to treat unilateral posterior canal BPPV. The results of the simulator showed excellent efficacy (100%) for the intended (posterior) canal. Surprisingly, the modified Epley maneuver also had excellent efficacy (100%) for the lateral canal and moderate efficacy (60%) for the anterior canal.

The Brandt–Daroff maneuver [11] was designed to treat bilateral posterior canal BPPV. The results of the simulator were surprising. Efficacy for the intended (posterior) canals was extremely poor (0%). In contrast, it showed excellent efficacy (100%) for the lateral canal and occasionally (20%) treated the anterior canal. The poor efficacy of the posterior canals is at variance with our clinical experience.

The Lempert roll [49] was designed to treat unilateral lateral canal BPPV. The results of the simulator showed excellent efficacy (100%) for the intended (lateral) canal and no effect (0%) for the posterior and anterior canals.

The Vannucchi–Asprella maneuver [50,51] was designed to treat unilateral lateral canal BPPV. Surprisingly, the results of the simulator showed very poor efficacy (10%) in the treatment of the lateral canal and similarly (10%) in the posterior and anterior canals. Regarding the results for the lateral canal, the main mechanism (7 out of 9 cases) of failure appeared to be that otoliths did not exit the ampulla.

One version of the Gufoni–Appiani maneuver [52] was designed to treat geotropic lateral canal BPPV. The results of the simulator showed excellent efficacy (100%) for the lateral canal. It occasionally (20%) treated the anterior canal and never (0%) treated the posterior canal.

The modified Zuma maneuver [53] was designed to treat unilateral geotropic lateral canal BPPV. The results of the simulator showed excellent efficacy (100%) for the intended (lateral) canal. It occasionally (30%) treated the anterior canal; in the other (70%) cases, otoliths refluxed from the anterior canal into the posterior canal. This maneuver never (0%) treated the posterior canal.

A second version of the Gufoni–Appiani maneuver [52,54] was designed to treat unilateral apogeotropic lateral canal BPPV. The results of the simulator showed moderate (50%) efficacy for the intended (lateral) canal and no efficacy (0%) for the posterior and anterior canals.

The Zuma maneuver [55] was designed to treat unilateral apogeotropic lateral canal BPPV. The results of the simulator showed excellent (100%) efficacy for the intended (lateral) canal. It rarely (10%) treated the anterior canal and never (0%) treated the posterior canal.

The Kurtzer hybrid maneuver [13] was designed to treat bilateral lateral canal BPPV. The results of the simulator were surprising. This maneuver had very poor (10%) efficacy for the intended (lateral) canal. It rarely (10%) treated the posterior canal and never treated the anterior canal. Otoliths in the posterior and anterior canals tended to mutually reflux in the simulation.

The Kim maneuver [56] was designed to treat unilateral anterior canal BPPV. The results of the simulator showed excellent (100%) efficacy for the intended (anterior) canal. Surprisingly, it also showed good efficacy (70%) for the lateral canal. It showed no efficacy (0%) for the posterior canal.

The modified (reversed) Semont maneuver [57] was designed to treat unilateral anterior canal BPPV. The results of the simulator showed excellent (100%) efficacy for the intended (anterior) canal. It rarely (10%) treated the lateral canal and never (0%) treated the posterior canal.

The reverse Epley maneuver [58,59] was designed to treat unilateral anterior canal BPPV. Surprisingly, the results of the simulator showed no efficacy (0%) for the intended (anterior) canal or any other canal.

The Rahko maneuver [60] was designed to treat unilateral anterior canal BPPV. The results of the simulator showed good (70%) efficacy for the intended (anterior) canal and no efficacy (0%) for the posterior and lateral canals.

The Garaycochea maneuver [61] was designed to treat unilateral anterior canal BPPV. The results of the simulator showed very good (80%) efficacy for the intended (anterior) canal and no efficacy (0%) for the posterior and lateral canals.

The Yacovino maneuver [12] and Casani maneuver [62] resemble each other. The results of the simulator showed good (70%) efficacy for the intended (anterior) canal; two of the three failed cases resulted from a canal conversion (otoliths refluxed into the posterior canal). These maneuvers had no efficacy for the posterior and lateral canals.

The Barreto maneuver [14] was designed to treat all three canals on one side. The results of the simulator showed excellent (100%) efficacy for the anterior canal, good efficacy (70%) for the posterior canal, and no efficacy (0%) for the lateral canal.

Some of these results from the simulator are surprising. For example, several maneuvers are quite effective at treating canals other than those for which they were designed, including the Epley maneuver, Foster maneuver, Gans maneuver, modified Epley maneuver, Brandt–Daroff maneuver, and Kim maneuver. In contrast, other maneuvers are poorly effective at treating the canal for which they were designed, including the Semont maneuver, Gans maneuver, Vannucchi–Asprella maneuver, Kurtzer hybrid maneuver, and reverse Epley maneuver.

Such discrepancies can arise from a variety of factors. First, the assumption that initial conditions are the “worst-case scenario” is probably not always true. Second, the simulator, with the limitations discussed earlier, may be inadequate. Third, most of the published maneuvers were designed and tested “freehand,” based on the treater’s estimate of position, rather than (for example) quantified angles measured by inertial motion units or predetermined by mechanical treatment chairs; studies have identified an astounding degree of variability in head positioning [63], suggesting inadequate human estimation of position angles.

Despite such discrepancies, these simulations appeared to show that a common mechanism for treatment failure was that a particular movement brought otoliths near the middle portion of a canal (which we will designate the “apex” below), but the next movement was large (typically one involving an excursion of ≥90°) and the canal’s new orientation allowed the otoliths to retreat toward the ampulla. Maneuvers using smaller excursions (such as 30° in the Kim maneuver or 45° in the Rahko maneuver) were less likely to exhibit this kind of failure. We kept this observation in mind when designing a new maneuver.

For this remainder of the discussion, we adopt the following terminology to refer to the different segments of the long arm of each canal:

- **Ampullary segment:** the segment of a semicircular canal closest to its ampule.
- **Crural segment** (called the “non-ampullary arm” in some of the literature): the segment of a semicircular canal closest to its crus (the singular crus for the horizontal canal; the common crus for the anterior and posterior canals).
- **Apex** (called the “vertex” in some of the literature): the segment of a semicircular canal between its ampullary segment and crural segment.

An otolith will move most readily when a canal plane is vertically oriented, and a line tangent to the canal lumen (where occupied by the otolith) is earth-vertical, as the vector of gravity is exerting maximum force. When a canal is oblique, the component of the gravitational vector collinear with the canal lumen (where occupied by the otolith) is diminished and thus less likely to overcome friction (between the canal wall and the otolith) and propel the otolith through the canal lumen.

When only a single canal is affected by BPPV, it is relatively easy to leverage gravity to manipulate those otoliths since only the orientation of that canal needs to be considered. However, if multiple canals are affected, then it becomes challenging to treat one canal while not interfering with the treatment of another.

In designing this maneuver, we applied several basic principles:

- We assume that the initial conditions are the “worst case scenario” in which the otoliths in each canal are in the ampullary segment (farthest away from the desired location).
- In any given position, otoliths should preferably advance toward the crus, or at least remain still, but not retreat toward the ampule. To avoid retreat toward the ampule, the plane of a given canal should be either vertical or oblique but never horizontal. However, if the initial conditions are not the “worst case scenario” mentioned above (for example, the otoliths are initially closer to the crural segment of a canal than to the ampullary segment), then the initial positions will provoke some “retreat” of canaliths toward the apex, but subsequent positions will coax them back toward the crural segment.
- Given the approximately orthogonal dispositions of the canal planes, in any given position, only one canal plane can be vertical, while the other two canal planes will be oblique. If two canals are vertical, then the third is horizontal, which risks permitting otoliths of that canal to retreat toward the ampule.

Many maneuvers involve some rotation of the head around its rostrocaudal axis (in other words, rotating the head in the yaw plane). Results of the simulator suggest that the larger the angle of such rotation required between two consecutive positions, the more likely the maneuver is to fail. There may be more than one reason for this, but probably the most important factor is simply that after moving from one position to another, it takes time for otoliths to settle into the most dependent portion of a canal [28,64] due to forces of friction (between the otoliths on the one hand, and the endolymphatic fluid and canal walls on the other hand); Peng and Qi summarize this as “The premise of a successful operation is that there is a long enough rest time between operations” [65]. The simulator by Teixido and colleagues [40,41] captures this behavior well.

Other factors, such as centrifugal force and inertia, likely play a less significant role. It may seem intuitive that any rotational head movement provokes a centrifugal force that would propel otoliths; however, in order for such centrifugal forces to be significant, the radius of the path must be very large. Significant centrifugal force is leveraged by maneuvers that involve the head moving along an arc whose rotation is centered around a distant site such as the hip, as occurs in the Gufoni maneuver [66] and Semont maneuver [67]. In contrast, centrifugal force is relatively insignificant when the head is rotating around its own vertical axis. As far as inertial forces are concerned, these probably have minimal effect on otolith movement [68].

Bearing in mind that a greater number of smaller intermediate movements (with pauses after each movement) permits otoliths to settle at the most dependent portion of the canal, we attempted to construct a sequence using intermediate positions to promote the gradual progression of otoliths in the desired direction through the canal toward the crus and lower the risk of otolith retreat toward the ampule. In practice, “breaking up” a large movement into a sequence of smaller positions (and requiring a pause in each position) is easier to execute than, for example, expecting a person to rotate the head 180° over the course of 120 seconds.

Below, we will display and discuss this maneuver—which we call the unilateral triple canal repositioning maneuver (UTCRM)—for the right ear. The mirror image of each position would be used for the left ear.

We adopt the following terminology for patient head/body positions:

- Side-lying position, as might be used during a supine roll test or logroll maneuver, with the rostrocaudal axis of the head parallel to the rostrocaudal axis of the trunk and the head’s mid-sagittal plane parallel to the surface of the earth. One could also substitute a position in which the patient is supine but with the head rotated 90° to the side (i.e., with the sagittal plane of the head parallel to the coronal plane of the trunk). For shorthand, we refer to this as the Lempert position in recognition of its use in the Lempert role [49].
- Oblique head-hanging position: position in which the patient is supine, with the neck extended (such that the head is hanging off the edge of the table) and the head turned 45° to one side (i.e., halfway between the sagittal plane and the coronal plane). For shorthand, we refer to this as the Dix–Hallpike position, a commonly used eponymous designation [69].
- The midline head-hanging position: the patient is supine, with the neck extended (such that the head is hanging off the edge of the table), and the head is midline. For shorthand, we refer to this as the Rose position [70,71,72].
- The penultimate position of this maneuver does not have an eponymous designation. It is a side-lying position, but with the patient’s neck laterally flexed (away from gravity) by 45° such that the uppermost ear is closer to the shoulder, similar to the penultimate position of the Rahko maneuver [60] or of the modified Epley maneuver [48]. Note that in this position, the common crus is nearly vertical, which helps prevent reflux into the anterior and posterior canals.

## 3. Results

The sequence of positions applied in the UTCRM for treating the right side are:

1. Neutral seated position.
2. Side-lying position (Lempert position) on the right side.
3. Oblique head-hanging position (Dix–Hallpike position) on the right side.
4. Midline head-hanging position (Rose position).
5. Oblique head-hanging position (Dix–Hallpike position) on the left side.
6. Side-lying position (Lempert position) on the left side.
7. Lie on the left side with the head angled upward (neck laterally flexed such that the right ear is inclined toward the right shoulder). One reason for including this intermediate position (rather than going directly to the neutral seated position listed below) is that otoliths near the apex of the posterior canal are more likely to be coaxed toward the crus.
8. Neutral seated position.

Figure 1, Figure 2, Figure 3, Figure 4, Figure 5, Figure 6, Figure 7 and Figure 8 depict the position of the patient’s head from various viewing angles and the starting and ending locations of the otoliths in each position.

Table 2 contains a description of each canal’s orientation in each position of the maneuver and the effect on the otoliths.

Video S1 progresses through each of the 8 positions of the maneuver. At each of the 8 positions, there are four video panels, each showing the BPPV Viewer simulator video generated from four positions (head of bed, foot of bed, right side of bed, left side of bed) to demonstrate as clearly as possible the movements of the otoliths in each canal.

The figures and videos of the head, labyrinths, and otoliths were generated from https://bppvviewer.com [40,41] (retrieved on 24 March 2025), which is freely available. The body simulations are from https://posemy.art (accessed 30 April 2025), which is freely available.

The results of the simulator showed 100% efficacy for all three canals. While this result is pleasing, it also merits skepticism since no treatment is perfect. It is safe to assume that this maneuver is imperfect and, in particular, that despite what we believe to be its advantageous design, there will be some undesirable otolith retreat toward the ampullae. This raises the question of whether repeating the maneuver would be feasible and, in particular, whether repeating the maneuver would cause “reflux” of the (previously) successfully treated otoliths. We explored this question by running simulations in which, after a first iteration of the maneuver, we introduced new otoliths into the ampullary segments of each canal (mimicking a situation in which there has been the complete retreat of some otoliths) and then conducted a second iteration of the maneuver. In 5 out of 5 simulation runs, the effects on the previously successfully treated otoliths were as follows. First, in the lateral canal, the previously (successfully) treated otoliths remained in the singular crus; they did not “reflux” into the lateral canal. Second, the previously (successfully) treated otoliths from the anterior and posterior canals “refluxed” from the common crus into the crural segment of the anterior canal in position 5 (left Dix–Hallpike); however, position 6 (left Lempert) moved those refluxed otoliths back toward the common crus, and position 7 (left side-lying with neck laterally flexed such that the uppermost ear is closer to the shoulder) brought them into the common crus. These simulator results suggest that the unilateral triple canal repositioning maneuver can be repeated and have a cumulative beneficial effect on any residual otoliths that had not been successfully treated by the previous iteration of the maneuver.

## 4. Discussion

The unilateral triple canal repositioning maneuver has these advantages:

1. For any given canal, its plane is vertically oriented at some point during this maneuver, offering the greatest likelihood for gravity to cause maximum transit of otoliths.
2. At other points in the maneuver, that canal plane is oblique, which may enable additional (desirable) progress of the otolith through the canal toward the crural segment but at least will prevent the (undesirable) retreat of otoliths toward the ampule.
3. Pausing at each intermediate position (breaking up the total movement) provides an opportunity for otoliths to settle in the most dependent portion of the canal, reducing the likelihood of otolith retreat.
4. In no position is any canal plane parallel with the earth’s surface. This reduces the risk of otolith retreat.

There are also several disadvantages of the unilateral triple canal repositioning maneuver. First, it is a relatively complex maneuver in terms of the number of positions. Second, it is necessary to know which side is involved. Third, if there is bilateral involvement, then this maneuver will only treat one side. Fourth, it does not address the type of BPPV known as the “short arm” [74] or “type 2” [75] variant, which usually affects the posterior canal.

The first problem (number of positions) merits comment. Omitting the initial and final positions (in which the patient is simply seated in a neutral position), the unilateral triple canal repositioning maneuver involves six intermediate positions. This is relatively complex compared to the number of intermediate positions of maneuvers summarized in Table 1, which, for the posterior canal, range from 2 to 5 positions; for the lateral canal, range from 2 to 6 positions; and for the anterior canal range from 2 to 6 positions. This characteristic is intentional for the reasons discussed earlier, namely, for maneuvers that leverage gravity to propel otoliths, a greater number of intermediate positions (each of which involves a smaller movement) gives otoliths a greater chance to settle into the most dependent position of the canal(s). However, the disadvantage is that a more complex maneuver introduces a greater chance for error in execution (whether by a clinician or a patient) and increases the likelihood of non-compliance (when a patient is performing it alone).

The second problem (necessity of knowing the involved side) bears further comment, as this lateralization is the main decision point in treatment because it dictates the choice of the side on which to apply the maneuver. Factors that a clinician may take into consideration include:

- While there is debate regarding the precise statistics of canals affected by BPPV, there is broad agreement that posterior canal BPPV is the most common variant of BPPV [76]. A patient with posterior canal BPPV will usually complain of symptoms when lying on or when rolling onto the affected side. Thus, if a patient describes symptoms typical of BPPV when lying on or when rolling toward a particular side, then it is reasonable to lateralize treatment (provisionally) to that side.
- BPPV usually (though not invariably) recurs on the same side, so if a patient has a prior documented history of BPPV on a particular side, then it is reasonable to lateralize treatment (provisionally) to that side.
- An ear affected by other diseases [77], such as labyrinthitis [78], vestibular neuritis [79], or Meniere’s disease [80], is more susceptible to developing BPPV. Therefore, if a patient who describes symptoms typical of BPPV also reports a prior history of another otologic disease on a particular side, then it is reasonable to lateralize treatment (provisionally) to that side.

The third problem (the possibility of bilateral involvement) also bears further comment. In any given position, the effect of gravity will have opposite effects on a pair of (approximately) co-planar canals; consequently, a position that tends to move otoliths in one canal toward its crus will also tend to move the otoliths of the contralateral co-planar canal toward its ampule. In fact, when we ran simulations with otoliths in all six canals, the result in 5 out of 5 simulation runs was that the otoliths in the three canals of the treated ear were successfully brought to the crura, whereas the otoliths in the three canals of the contralateral ear almost always ended up in or near the ampullae. This has two consequences that are relevant to the unilateral triple canal repositioning maneuver. First, if a patient has unilateral BPPV (in any combination of canals) and a practitioner treats the incorrect side with the unilateral triple canal repositioning maneuver, then the patient will remain symptomatic (or even become increasingly symptomatic) after the maneuver (because all of the otoliths in the affected canals will be at or near the ampullae); such an outcome would be an indication to switch treatment sides. Second, this maneuver will fail (at least initially) when there is bilateral involvement. Bilateral BPPV is relatively uncommon, occurring in about 11% of cases [80]. In such cases, it would be reasonable to treat one side over the course of several days to try to resolve BPPV on that side and only thereafter switch to treating the contralateral side.

The fourth problem (“short arm” or “type 2” BPPV), while worth considering, is a risk for any maneuver since all maneuvers aim to bring otoliths into the utricle, and thus, all have the potential to permit otoliths to enter the short arm of various canals (usually the posterior canal); therefore, this potential complication is shared by all maneuvers and is not unique to the unilateral triple canal repositioning maneuver.

## 5. Conclusions

We began by analyzing the results of applying a BPPV simulator to several published maneuvers. By drawing from those observations, by using several design principles, and with the aid of a BPPV simulator, we have designed a canalith repositioning maneuver to treat all three canals on one side simultaneously. If clinically verified, this should offer the advantages discussed earlier, simplifying the diagnostic and therapeutic process.

While we believe that the BPPV simulator by Teixido and colleagues [40,41] is an excellent tool and that our design principles are reasonable, the unilateral triple canal repositioning maneuver that we have proposed remains theoretical, with all its attendant limitations. This maneuver should be studied empirically as a standalone maneuver. It would also be desirable to compare its real-life efficacy to that of other established maneuvers. 

## Figures and Tables

**Figure 1:**
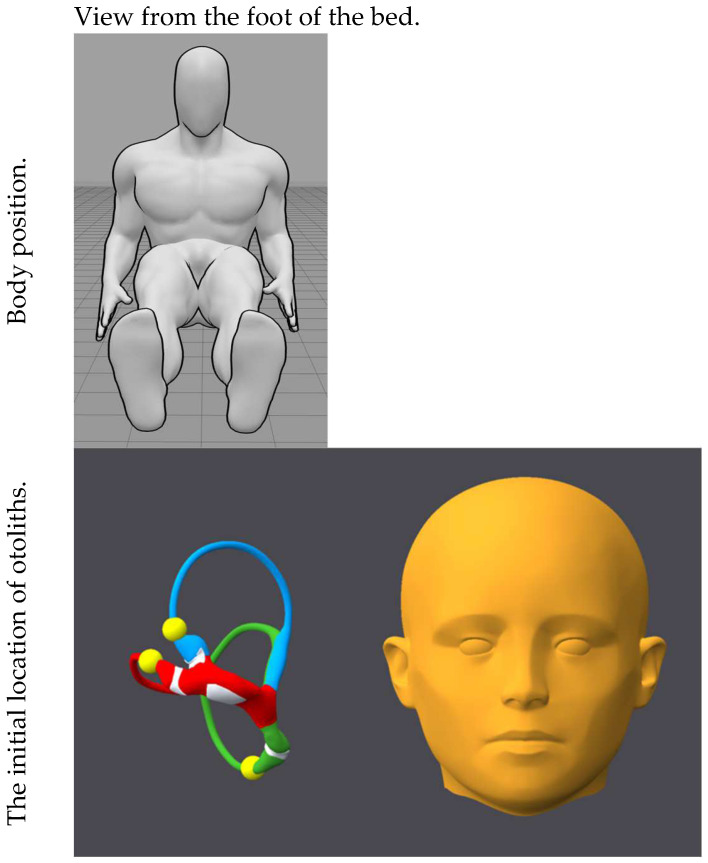
Position 1, seated (neutral), viewed from the foot of the bed, with otolith locations. The horizontal canal is colored red; the posterior canal is colored green; the anterior canal is colored blue; the otoliths are colored yellow.

**Figure 2:**
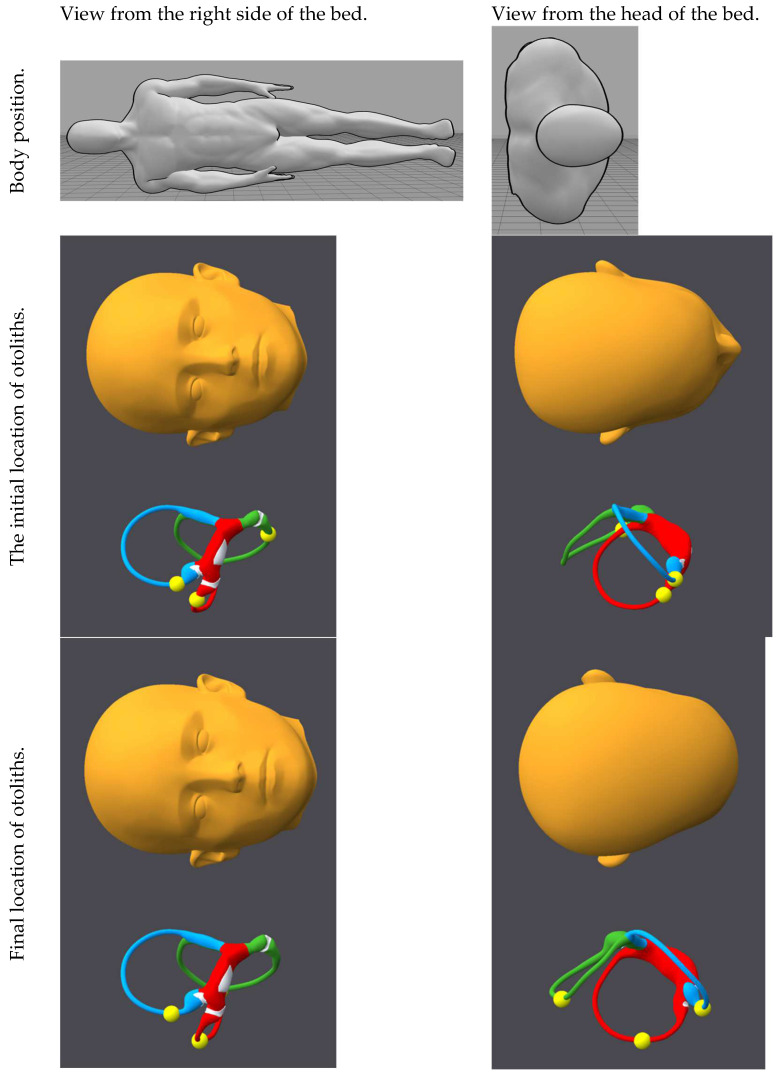
Position 2, right Lempert, viewed from the right side of the bed and from the head of the bed, with otolith locations at the beginning and end of this position. The horizontal canal is colored red; the posterior canal is colored green; the anterior canal is colored blue; the otoliths are colored yellow.

**Figure 3:**
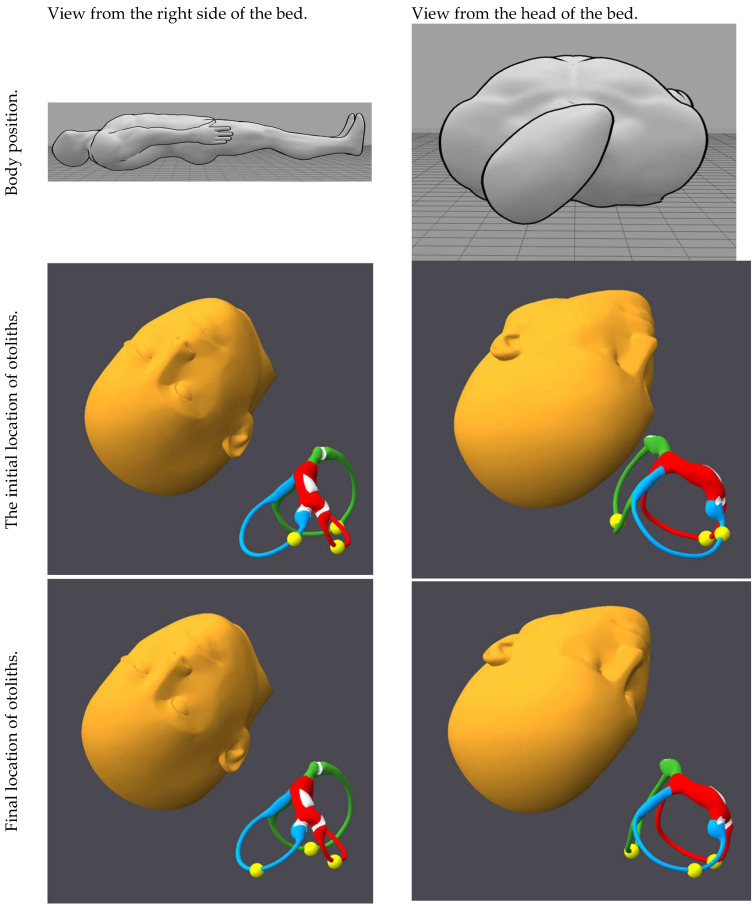
Position 3, right Dix–Hallpike, viewed from the right side of the bed and head of the bed, with otolith locations at the beginning and end of this position. The horizontal canal is colored red; the posterior canal is colored green; the anterior canal is colored blue; the otoliths are colored yellow.

**Figure 4:**
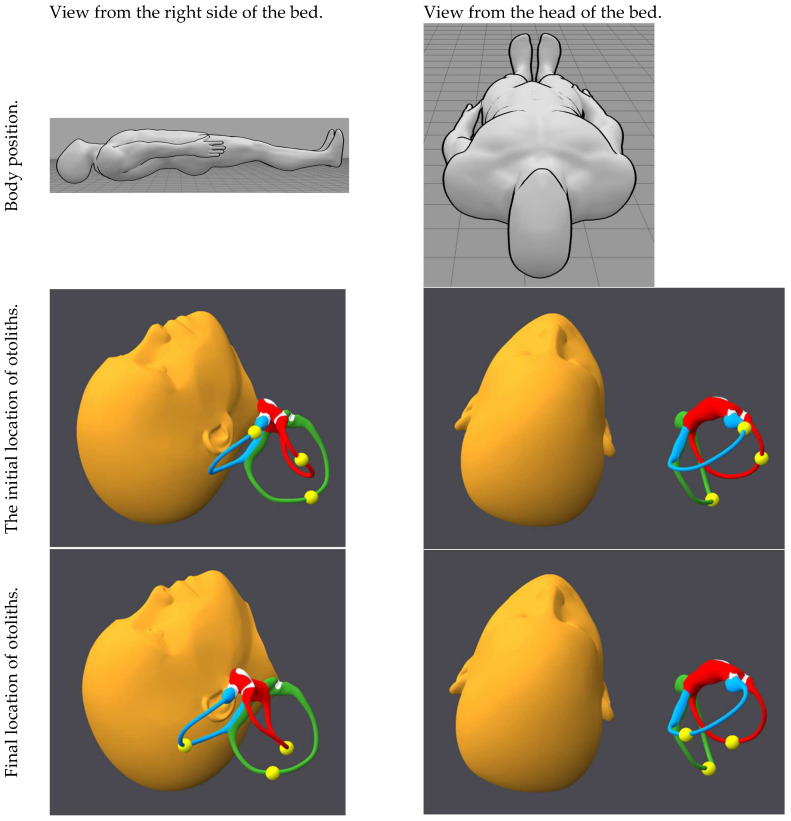
Position 4, Rose position, viewed from the right side of the bed and the head of the bed, with otolith locations at the beginning and end of the position. The horizontal canal is colored red; the posterior canal is colored green; the anterior canal is colored blue; the otoliths are colored yellow.

**Figure 5:**
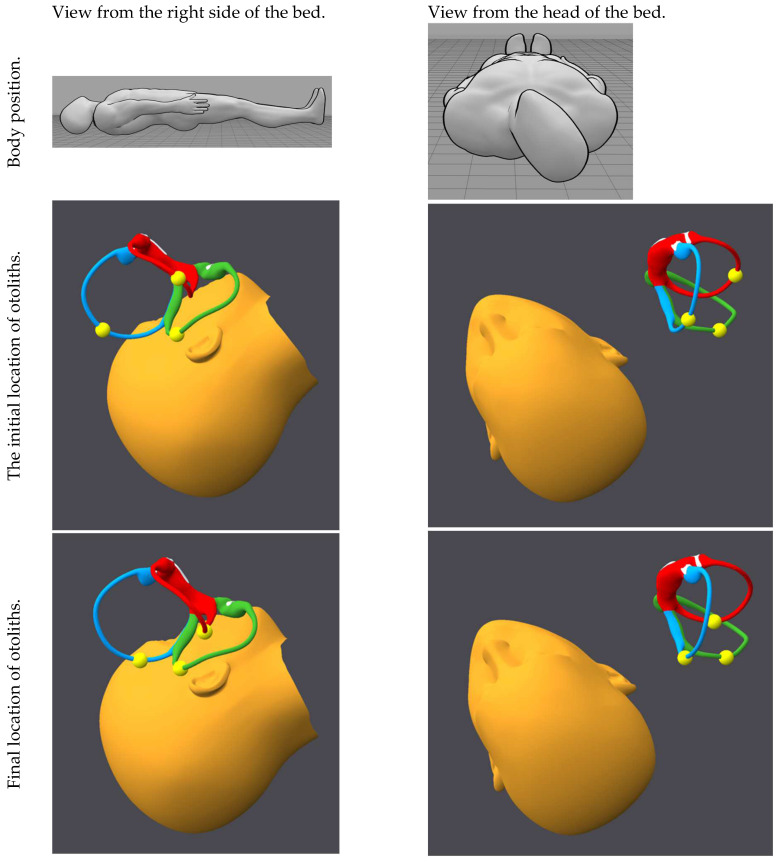
Position 5, left Dix–Hallpike, viewed from the right side of the bed and the head of the bed, with otolith locations at the beginning and end of the position. The horizontal canal is colored red; the posterior canal is colored green; the anterior canal is colored blue; the otoliths are colored yellow.

**Figure 6:**
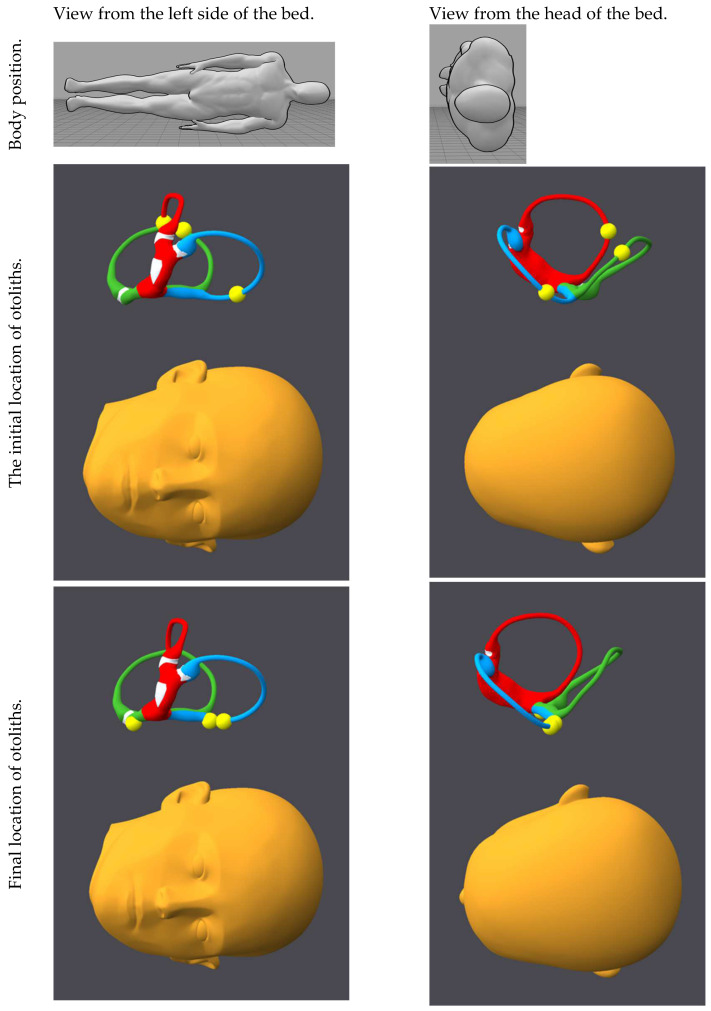
Position 6, left Lempert, viewed from the left side of the bed and the head of the bed, with otolith locations at the beginning and end of the position. The horizontal canal is colored red; the posterior canal is colored green; the anterior canal is colored blue; the otoliths are colored yellow.

**Figure 7:**
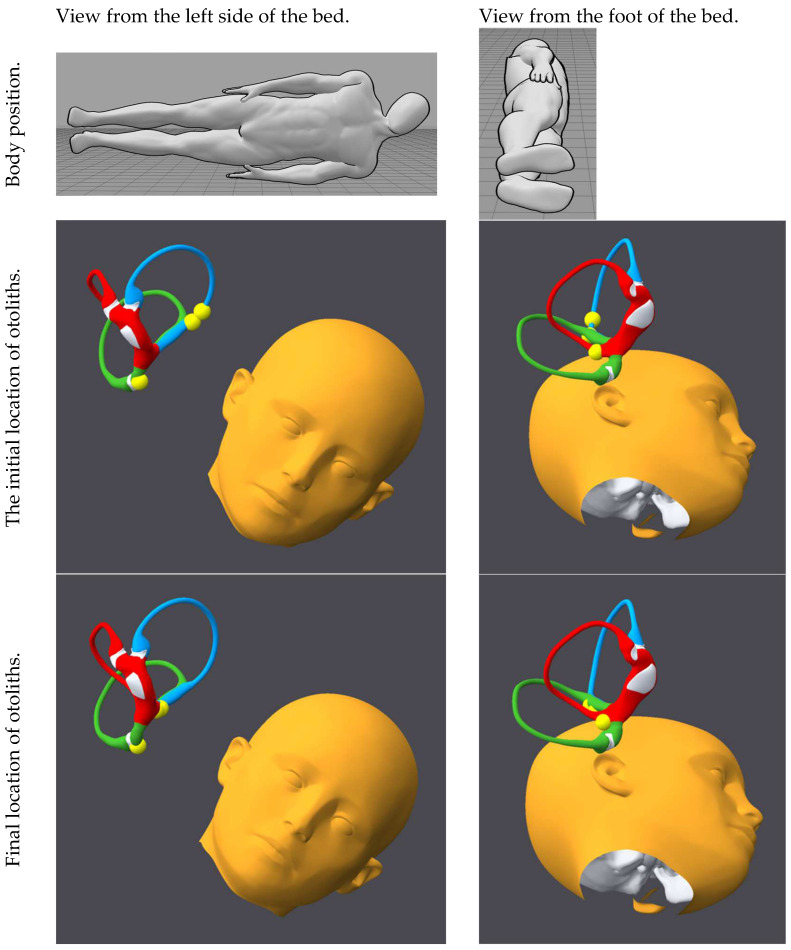
Position 7, left lateral decubitus with head angled upward, viewed from the left side of the bed and the foot of the bed, with otolith locations at the beginning and end of the position. The horizontal canal is colored red; the posterior canal is colored green; the anterior canal is colored blue; the otoliths are colored yellow.

**Figure 8:**
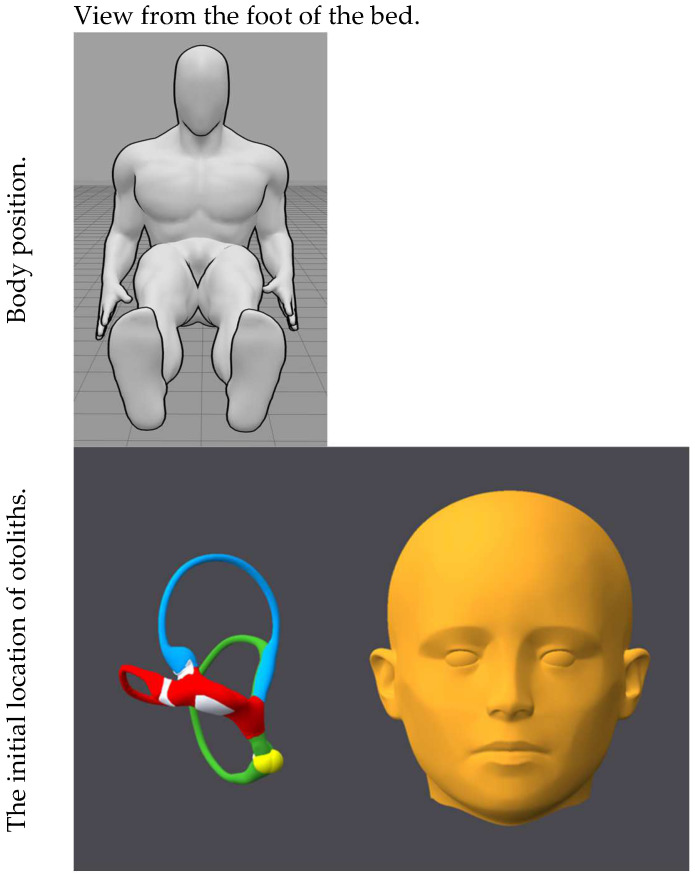
Position 8, seated (neutral), viewed from the foot of the bed, with otolith positions. The horizontal canal is colored red; the posterior canal is colored green; the anterior canal is colored blue; the otoliths are colored yellow.

**Table 1:** Results of BPPV Viewer simulation on a selection of published maneuvers. Abbreviations: PC = posterior canal; LC = lateral canal; AC = anterior canal.

Maneuver	Number of Positions	Posterior Canal (PC)		Lateral Canal (LC)		Anterior Canal (AC)	
		PC Success	PC Failure	LC Success	LC Failure	AC Success	AC Failure
Maneuvers intended to treat unilateral posterior canal							
**Epley**. Excellent efficacy for all canals.	3	10 Otoliths usually reflux into AC before resolving.	0	10	0	9	1 Occasionally, otoliths fail to exit the ampulla.
**Semont**. Good efficacy for PC. Occasionally treats LC.	2	3	7 Usually, in position 2, otoliths reflux into AC and then remain there. Sometimes, in position 1, otoliths reach the apex, but then in position 2, they retreat.	1	9 In position 1, otoliths reach the apex, but then in position 2, they usually retreat.	0	10 In position 1, the AC plane remains horizontal, so otoliths remain stationary, not even advancing toward the apex.
**Semont plus**. Very good efficacy for PC. Occasionally treats AC.	2	8	2 Sometimes (1) refluxes into AC. Sometimes (1) retreats from the apex.	0	10 Retreats from apex.	3	7 Retreats from the apex or does not reach the apex.
**Foster**. Very good efficacy for PC. Good efficacy for AC. Rarely treats LC.	5	8 May (7) transiently reflux into AC.	2	1	9 Retreats from apex.	6	4 Does not progress from the ampulla.
**Gans**. Modest efficacy for PC. Very good efficacy for LC. Good efficacy for AC.	3	2	8 Retreats from apex.	10	0	8 Otoliths sometimes (1) reflux into PC and then resolve.	2 Otoliths sometimes (1) reflux into PC and then remain there.
**Modified Epley**. Excellent efficacy for PC and LC. Good efficacy for AC.	4	10	0	10	0	6	4 Does not exit the ampulla.
Maneuvers intended to treat bilateral posterior canals							
**Brandt–Daroff**. Excellent efficacy for LC. Occasionally treats AC.	3	0	10	10	0	2	8 Usually retreats from the apex.
Maneuvers intended to treat unilateral lateral canal (geotropic or apogeotropic)							
**Lempert roll**. Excellent efficacy for LC.	4	0	10	10	0	0	10 In position 2, otoliths from AC often (7) reflux into PC and then remain there.
**Vannucchi–Asprella**. Poor efficacy for all canals.	3	1 Sometimes (1) refluxes into AC, then resolves.	9 Approaches but then retreats from the apex.	1	9 In some instances (7), otoliths fail to exit the ampulla.	1	9
Maneuvers intended to treat unilateral lateral canal, geotropic variant							
**Gufoni–Appiani, geotropic**. Excellent efficacy for LC. Rarely treats AC.	2	0	10	10	0	2 Rarely do otoliths escape the AC cupula during the first position.	8
**Modified Zuma**. Excellent efficacy for LC. Modest efficacy for AC.	5	0	10	10	0	3	7 Sometimes (4) otoliths reach the common crus but reflux into the PC.
Maneuvers intended to treat unilateral lateral canal, apogeotropic variant							
**Gufoni–Appiani, apogeotropic**. Modest efficacy for LC alone.	3	0	10	5	5	0	10
**Zuma**. Excellent efficacy for LC alone. Rarely treats AC.	5	0	10	10	0	1	9 Sometimes (5) in position 2, AC refluxes into PC and remains there.
Maneuvers intended to treat bilateral lateral canals							
**Kurtzer hybrid**. Poor efficacy overall.	6	1	9 Otoliths sometimes alternate PC and AC, mutually refluxing	1	9 Often, otolith remains in the ampulla.	0	10 Otoliths sometimes alternate PC and AC, mutually refluxing
Maneuvers intended to treat unilateral anterior canal							
**Kim**. Excellent efficacy for AC, modest efficacy for LC.	4	0	10	7	3	10	0
**Reverse Semont**. Excellent efficacy for AC. Rarely treats LC.	2	0	10	1	9	10	0
**Reverse Epley**. No efficacy.	3	0	10	0	10	0	10
**Rahko**. Good efficacy for AC alone.	3	0	10	0	10	7	3 Sometimes, otoliths fail to exit the ampulla.
**Garaycochea**. Very good efficacy for AC alone.	6	0	10	0	10	8	2
Maneuvers intended to treat bilateral anterior canals							
Yacovino. Good efficacy for AC alone.	2	0	10	0	10	7	3 Sometimes (2) otoliths reflux from AC to PC and remain there. Sometimes (1) otoliths fail to exit the ampulla.
Maneuvers intended to treat multiple canals							
Barreto. Excellent efficacy for AC, moderate efficacy for PC, and no efficacy for LC.	2	7 Sometimes (2) refluxes into AC before resolving	3 Sometimes (3) otoliths do not exit the ampulla.	0	10	10	0

**Table 2:** Sequence of positions for the right-sided unilateral triple canal repositioning maneuver, with commentary on the effect each position has in each canal. The colors correspond to the canals in the Supplementary Video S1 demonstrating the simulation of the maneuver.

Position	Right Anterior Canal	Right Posterior Canal	Right Horizontal Canal
1. Neutral (seated).	The canal is vertical, with the apex superior to the ampullary segment and crural segment. Canalith is at the proximal part of the ampullary segment.	The canal is vertical, with the apex superior to the crural segment and ampullary segment. Canalith is at the proximal part of the ampullary segment.	The canal is nearly horizontal, with the ampullary segment slightly superior to the apex and superior to the crural segment. Canalith is at the proximal part of an ampullary segment.
2: Right Lempert (side-lying). Acts predominantly on the right horizontal canal.	The canal is oblique, with the crural segment superior to the apex and superior to the ampullary segment. Canalith moves slightly along the ampullary segment toward the apex.	The canal is oblique, with an ampullary segment and a crural segment superior to the apex. Canalith moves from the ampullary segment toward the apex.	The canal is nearly vertical, with the crural segment superior to the ampullary segment superior to the apex. Canalith moves from the ampullary segment to the apex.
3: Right Dix–Hallpike. Acts predominantly on the right posterior canal.	The canal is oblique, with an ampullary segment and a crural segment superior to the apex. Canalith reaches an apex.	The canal is vertical, with the ampullary segment superior to the crural segment superior to the apex. Canalith reaches an apex.	The canal is oblique, with an ampullary segment and a crural segment superior to the apex. Canalith reaches the apex.
4: Rose position.	The canal is oblique, with the ampullary segment superior to the apex and superior to the crural segment. Canalith progresses along the apex.	The canal is oblique, with ampullary segment and crural segment superior and apex inferior. Canalith progresses along the apex.	The canal is oblique, with an ampullary segment and a crural segment superior to the apex. Canalith progresses along the apex.
5: Left Dix–Hallpike. Acts predominantly on the right anterior canal.	The canal is vertical, with the ampullary segment superior to the crural segment superior to the apex. Canalith progresses along the apex.	The canal is oblique, with the ampullary segment superior to the crural segment superior to the apex. Canalith progresses along the apex.	The canal is oblique, with the ampullary segment superior to the crural segment superior to the apex. Canalith progresses along the apex.
6: Left Lempert (side-lying). Acts predominantly on the right horizontal canal.	The canal is oblique, with the ampullary segment superior to the apex and superior to the crural segment. Canalith moves slightly toward common crus.	The canal is oblique, with the apex superior to both the ampullary segment and the crural segment. Canalith progresses toward common crus.	The canal is nearly vertical, with the apex superior to the ampullary segment and superior to the crural segment. Canalith progresses toward singular crus.
7: Left lateral decubitus, with neck laterally flexed by 45° such that the upper ear is closer to the shoulder.	The canal is oblique, with the apex superior to the ampullary segment and crural segment. Canalith progresses toward or into common crus.	The canal is oblique, with the apex superior to the crural segment and superior to the ampullary segment. Canalith progresses toward or into common crus.	The canal is oblique, with the ampullary segment superior to the apex and superior to the crural segment. Canalith progresses into a singular crus.
8: Neutral (seated).	The canal is vertical, with the apex superior to the ampullary segment and superior to the crural segment. Canalith progresses through common crus into the utricle.	The canal is vertical, with the crural segment superior to the apex superior to the ampullary segment. Canalith progresses through common crus into the utricle.	The canal is slightly oblique, with the ampullary segment superior to the apex and superior to the crural segment. Canalith progresses through a singular crus into the utricle.

## Data Availability

The original contributions presented in this study are included in the article/Supplementary Material. Further inquiries can be directed to the corresponding author.

## Supplementary Materials

The following supporting information can be downloaded at: https://www.mdpi.com/article/10.3390/audiolres15030055/s1, Video S1: Video demonstration of simulation of the unilateral triple canal repositioning maneuver.
